# Geographic variation in shortfalls of dementia specialists in the United States

**DOI:** 10.1093/haschl/qxae088

**Published:** 2024-07-18

**Authors:** Jodi L Liu, Lawrence Baker, Annie Yu-An Chen, Jue (Jessie) Wang

**Affiliations:** RAND, Economics, Sociology, and Statistics Department, Santa Monica, CA 90407-2138, USA; RAND, Pardee RAND Graduate School, Santa Monica, CA 90407-2138, USA; RAND, Pardee RAND Graduate School, Santa Monica, CA 90407-2138, USA; RAND, Economics, Sociology, and Statistics Department, Boston, MA 02116, USA

**Keywords:** dementia, Alzheimer's, healthcare workforce

## Abstract

Dementia specialists—neurologists, geriatricians, and geriatric psychiatrists—serve a critical clinical function in diagnosing early-stage Alzheimer's disease and determining eligibility for treatment with disease-modifying therapies. However, the availability of dementia specialists is limited and varies across the United States. Using data from the Area Health Resources Files, we found that the median density of dementia specialists across hospital referral regions in United States is 28.8 per 100 000 population aged 65 years and older (interquartile range 19.3-43.6). We derived thresholds of 33-45 dementia specialists per 100 000 population aged 65 years and older as the provider density necessary to care for older adults with mild cognitive impairment and dementia. Based on these thresholds, we estimated that 34%-59% of the population aged 65 years and older resided in areas with potential dementia specialist shortfalls. The extent of potential shortfalls varied by state and rurality. A better understanding of potential gaps in the availability of dementia specialists will inform policies and practices to ensure access to services for people with cognitive impairment and dementia.

## Introduction

The Food and Drug Administration traditional approval of lecanemab and donanemab for early-stage Alzheimer's disease (AD) and a robust pipeline of AD-modifying therapies in clinical trials bring urgency to the question of how patients will access AD-modifying therapies.^1-4^ Unlike dementia medications used for symptom management, these therapies slow the disease progression and eligible patients must be diagnosed with early-stage AD—mild cognitive impairment (MCI) due to AD or mild Alzheimer's dementia. However, undiagnosed dementia is common, with early-stage AD more frequently undetected.^5-7^ As this class of therapies continues to emerge, it is critical to improve the underdiagnosis of early-stage AD.

Although early detection and clinical assessment may be performed by primary care providers (PCPs) and neuropsychologists, it is typically neurologists, geriatricians, and geriatric psychiatrists who would diagnose early-stage AD, confirm the Alzheimer's pathology, and recommend and monitor treatment.^8,9^ Shortages of these specialists have been acknowledged, but studies have typically focused on a single specialty and older adults generally (eg, age 65 years and older) without specific consideration of early-stage AD.^10-13^ Prior research estimated that shortfalls of dementia specialists could result in long wait times and delayed treatment.^14,15^ Access to care is likely to be unequal across the United States as estimated neurologist and geriatrician shortfalls vary by state.^12,16^ In addition, poorer access to specialists in rural areas contributes to higher rates of preventable hospitalization and mortality.^17^

To better understand the geographic variation in dementia specialists, we examined the density of dementia specialists as the number of neurologists, geriatricians, and geriatric psychiatrists per population aged 65 years and older in hospital referral regions (HRRs) in the United States and the share of the population residing in areas with potential shortfalls.

## Data and methods

To estimate the number of dementia specialists, we used 2020 county-level data from the 2021-2022 Area Health Resources Files. We identified neurologists, general internal medicine physicians, family medicine physicians, and psychiatrists involved in patient care. We estimated the proportions of geriatricians in internal and family medicine and geriatric psychiatrists in psychiatry based on state-level data where available^18,19^ or national data.^19,20^ We used county-level population data from the 2020 Census.

We computed the specialist density for HRRs, which are geographic regions representing healthcare markets for tertiary care and defined as areas with at least one hospital performing major cardiovascular procedures and neurosurgery. Contained within the 306 HRRs are 3436 hospital service areas that represent local healthcare markets.^21^ We used county-to-ZIP code and ZIP-to-HRR crosswalks from the Dartmouth Atlas and calculated the number of dementia specialists per 100 000 population aged 65 years and older in each HRR. We used the 2013 Rural/Urban Continuum Codes to categorize counties, with 1-3 as urban and 4-9 as rural.^22^

To define target density thresholds, we first considered people with MCI, who may have early-stage AD. Assuming 22.9% MCI prevalence^23^ and a 700-patient panel,^16^ we estimated 33 dementia specialists per 100 000 population aged 65 years and older (0.229/700*100 000) would be needed. For the second threshold, we considered those with MCI or dementia (prevalence 8.5%^23,24^) and estimated 45 dementia specialists per 100 000 population aged 65 years and older ((0.229 + 0.085)/700*100 000).

There were several limitations to this analysis. First, the providers and populations included were not perfectly aligned with those involved in AD care. Healthcare workforce shortages are frequently defined by provider density or population to provider ratios;^13,25^ however, there are not definitive thresholds for dementia care. Although neurologists are generally able to diagnosis AD, behavioral neurology is a small subspecialty and not all neurologists assess cognitive impairment. Excluding neurologists who would not typically conduct cognitive assessments would increase the estimated shortfall. Although AD prevalence is low under age 65 years, including younger populations would somewhat increase the estimated shortfall. Second, disease prevalence and provider information were not available at the HRR level. We used national estimates of MCI and dementia prevalence and state (where available) and national data on the number of providers by specialty. Third, we did not account for people traveling outside their HRR of residence for care. While people do cross boundaries for care, perhaps more frequently in rural areas, movement across HRRs for care is relatively low.^26^ This results in portraying geographic disparities that could be larger than actual differences in access. However, this may be counterbalanced by other barriers such as financial and transportation difficulties. Fourth, we did not consider any PCPs as dementia-proficient practitioners. There are primary care-led models in which PCPs perform clinical assessments and AD diagnosis; however, these activities are infrequent at typical primary care practices. Finally, this analysis is descriptive and does not consider causation. The presence of healthcare professionals in a geographic area is one measure of availability of services, which is related to access to care. Further analyses are needed beyond this HRR-level analysis of dementia specialists to better measure access to dementia care.

## Results

**Figure 1.**
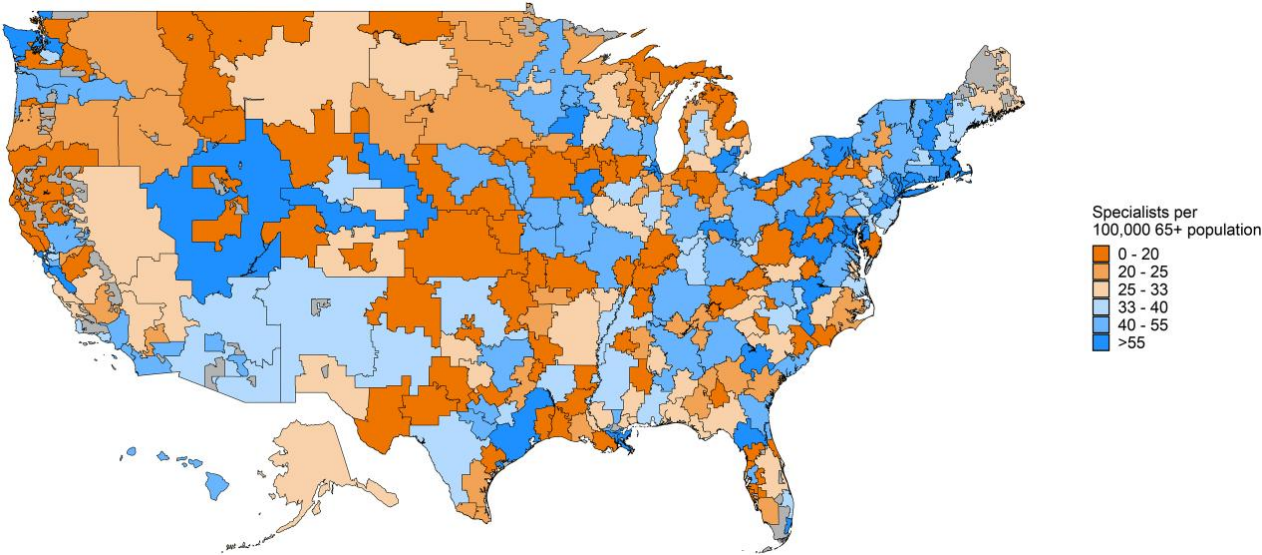
Dementia specialists per 100 000 population aged 65 years and older in HRRs, 2020. Area Health Resources Files (AHRF), 2021-2022; U.S. Census Bureau, 2020. Values are not shown for areas that are not defined within a hospital referral region (HRR).

The median density of dementia specialists was 28.8 per 100 000 population aged 65 years and older (interquartile range 19.3-43.6) across HRRs (Figure 1). Of the 306 HRRs, we found 178 HRRs (58%) had fewer than 33 dementia specialists per 100 000 population aged 65 years and older, and 234 HRRs (76%) had fewer than 45. The first density threshold represents the number of specialists needed for patients with MCI, while the second represents the level needed for patients with MCI or dementia. The density of dementia specialists by state is shown in Appendix Figure A.1.

Of the 55.7 million adults aged 65 years and older, we found that 18.8 million (33.9%) resided in areas with fewer than 33 dementia specialists per 100 000 population aged 65 years and older (Table 1). About 33.1 million (59.4%) resided in areas with fewer than 45 dementia specialists per 100 000 population aged 65 years and older. Although the urban population was larger than the rural population, a larger share of rural populations resided in shortfall areas. Approximately 25.7 million or 55.6% of the urban population aged 65 years and older were in shortfall areas, whereas 7.4 million or 78.5% of the rural population age 65 years and older were in shortfall areas.

**Table 1.** Population residing in areas by dementia specialist density.

Dementia specialists per 100 000 population aged 65 years and older	Population aged 65 years and older, millions (%)	Urban population aged 65 years and older, millions (%)	Rural population aged 65 years and older, millions (%)
9-20	7.2 (12.9%)	4.8 (10.3%)	2.4 (25.5%)
20-33	11.7 (21.0%)	9.2 (19.8%)	2.5 (26.7%)
33-45	14.2 (25.6%)	11.8 (25.4%)	2.5 (26.2%)
45 and greater	22.6 (40.6%)	20.6 (44.4%)	2.0 (21.5%)

Area Health Resources Files (AHRF), 2021-2022; U.S. Census Bureau, 2020.

## Discussion

We found substantial variation in the density of dementia specialists across HRRs and states. More than half of HRRs had shortfalls of dementia specialists. There was overall a higher share of the rural population in shortfall areas, but the extent to which shortfalls affected urban and rural populations varied by state (Appendix Figure A.2).

Other work has documented geographic, socioeconomic, and racial disparities in dementia care.^27^ For example, people living with dementia in rural areas had fewer physician visits, higher use of nursing facility care, and higher mortality rates compared to those in urban areas.^28,29^ People in rural areas were less likely to receive neuropsychological testing; however, they may be more likely to visit PCPs for diagnosis and symptom management,^30^ which suggests potential for more training or guidance to PCPs to help alleviate the limited availability of specialists. Telehealth models such as Project ECHO (Extension for Community Healthcare Outcomes) that connect PCPs and specialists to provide education and support could help improve access in rural areas.^31,32^

Limited availability of dementia specialists could hinder timely access to diagnosis, which delays care planning, financial planning, and possible treatment with disease-modifying therapies that are time-critical to slow disease progression. Increasing dementia proficiency among PCPs, shifting parts of the detection and diagnostic processes to the primary care setting, establishing more collaborative care models involving PCPs and non-physician staff as well as routine workflows would help reduce the need for specialist care.^33,34^ Timely detection and diagnosis of early-stage AD will require collaboration between PCPs and dementia specialists, as well as other stakeholders including patients and families, health system leaders, payers, and public health officials.

## Conclusion

Approximately one-third to nearly 60% of the population aged 65 years and older lived in areas with potential shortfalls of dementia specialists. The density of dementia specialists varied across HRRs, states, and urban versus rural areas. Given developments in early detection and new therapeutics for early-stage AD, a better understanding of potential gaps in the availability of dementia specialists will inform policies and practices necessary to ensure access to services for people with cognitive impairment and dementia.

## Supplementary Material

qxae088_Supplementary_Data
