# Supplementary material for: Geographic variation in shortfalls of dementia specialists in the United States
Source: Health Aff Sch. 2024 Jul 18;2(7):qxae088. doi: 10.1093/haschl/qxae088 (PMC11288326; doi:10.1093/haschl/qxae088)
Supplement: qxae088_Supplementary_Data [file qxae088_supplementary_data.zip › Appendix.docx]

**APPENDIX**

**Supplemental Results**

The density of dementia specialists varied by state, from 14.3 dementia specialists per 100,000 population aged 65 and older in Wyoming to 151.1 in Washington D.C. (Figure A.1). Fifteen states had fewer than 33 dementia specialists per 100,000 population aged 65 and older, and 36 states had fewer than 45.

The share of the population residing in areas with shortfalls of dementia specialists also varied by state and rurality. For example, all shortfall areas in Vermont were rural, all shortfall areas in Delaware and New Jersey were urban; most states had a range of urban and rural shortfall areas (Figure A.2).

**Figure A.1.** Dementia Specialist per 100,000 Population Aged 65 and Older by State, 2020

SOURCE: Area Health Resources Files (AHRF), 2021–2022; U.S. Census Bureau, 2020

NOTE: The vertical line indicates the national average of 44.5 specialists per 100,000 population aged 65 and older.

**Figure A.2.** Percentage of the Population with Shortfalls in Rural Versus Urban Areas, by State

SOURCE: Area Health Resources Files (AHRF), 2021–2022; U.S. Census Bureau, 2020
